# Hematological predictive markers for recurrent or metastatic squamous cell carcinomas of the head and neck treated with nivolumab: A multicenter study of 88 patients

**DOI:** 10.1002/cam4.3124

**Published:** 2020-05-22

**Authors:** Takashi Matsuki, Isaku Okamoto, Chihiro Fushimi, Michi Sawabe, Daisuke Kawakita, Hiroki Sato, Kiyoaki Tsukahara, Takahito Kondo, Takuro Okada, Yuichiro Tada, Kouki Miura, Go Omura, Taku Yamashita

**Affiliations:** ^1^ Department of Otorhinolaryngology, Head and Neck Surgery Kitasato University School of Medicine Sagamihara Japan; ^2^ Department of Otorhinolaryngology, Head and Neck Surgery Tokyo Medical University School of Medicine Tokyo Japan; ^3^ Department of Head and Neck Oncology and Surgery International University of Health and Welfare Mita Hospital Tokyo Japan; ^4^ Department of Otorhinolaryngology, Head and Neck Surgery Nagoya City University Graduate School of Medical Sciences Nagoya Japan; ^5^ Department of Otorhinolaryngology, Head and Neck Surgery Tokyo Medical University Hachioji Medical Center Hachioji Japan; ^6^ Department of Head and Neck Oncology National Cancer Center Hospital Tokyo Japan

**Keywords:** biomarkers, head and neck cancer

## Abstract

**Background:**

There is increasing evidence that immunotherapy with nivolumab, an anti‐programmed death 1 monoclonal antibody, is effective in the treatment of recurrent or metastatic squamous cell carcinoma of the head and neck (R/M SCCHN). However, the predictive role of hematological inflammatory markers such as neutrophil‐to‐lymphocyte ratio (NLR) and the modified Glasgow prognostic score (mGPS) in patients with R/M SCCHN treated with nivolumab remains unclear.

**Methods:**

We conducted a multi‐institutional cohort study to evaluate the impact of pretreatment NLR and mGPS on overall survival (OS) and progression‐free survival (PFS) in patients with R/M SCCHN treated with nivolumab in Japan. From 2012 to 2013, 102 patients were eligible, of whom 88 were finally included in the analysis. mGPS was calculated as follows: mGPS of 0, C‐reactive protein (CRP) ≤1.0 mg/dL; 1, CRP > 1.0 mg/dL; and 2, CRP > 1.0 mg/dL and albumin < 3.5 mg/dL. Optimal cutoff point of dichotomized NLR was calculated using the area under the receiver operating characteristic curve (AUROC). Hazard ratios (HRs) and 95% confidence intervals (95% CIs) were estimated by Cox proportional hazard models adjusted by potential confounders.

**Results:**

Higher NLR was significantly associated with worse survival (1‐year OS: 45.3% vs 16.3%, log‐rank *P*‐value < .001, adjusted HR: 4.40 (95% CIs: 1.78‐10.88); one‐year PFS: 39.1% vs 9.0%, *P*‐value = .001, adjusted HR: 3.37 (95% CI: 1.64‐6.92)). In addition, high mGPS (=2) was significantly associated with worse survival compared to low mGPS (=0) (1‐year OS: 37.4% vs 26.1%, *P*‐value = .004, adjusted HR: 4.20 (95% CI:1.54‐11.49); 1‐year PFS: 41.5% vs 24.8%, *P*‐value = .007, adjusted HR: 2.01 (95% CI: 0.87‐4.68)). These associations were consistent with subgroup analyses stratified by potential confounders.

**Conclusions:**

Pretreatment NLR and mGPS might be predictive markers of survival in patients with R/M SCCHN treated with nivolumab.

## Graphical abstract

Immunotherapy with nivolumab is effective in the treatment of R/M SCCHN. However, reliable predictive markers have remained unclear. Higher pre‐treatment NLR and mGPS were significantly associated with worse survival, suggesting that these markers may be predictors of treatment with nivolumab.

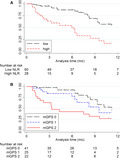

## 1 INTRODUCTION

Patients with recurrent or metastatic squamous cell carcinoma of the head and neck (R/M SCCHN) have a poor prognosis and experience severe problems in QOL arising from localization of the tumor. Platinum‐based chemotherapies are frequently used for patients with R/M SCCHN. Despite palliative treatment, however, severe adverse events are not uncommon, including conditions such as renal dysfunction and bone marrow suppression, and many patients require hospitalization.

Nivolumab, an anti‐programmed death 1 (PD‐1) monoclonal antibody, is an immune checkpoint inhibitor (ICI) that binds to PD‐1 and blocks signaling mediated by PD‐1/PD‐L1 interactions. After its efficacy was demonstrated in a phase 3 trial in patients with R/M SCCHN,^1^ it was approved for R/M SCCHN after platinum drug administration in Japan from March 2017. Further evidence of efficacy has been obtained since then.^2^, ^3^, ^4^ Despite the high expense of treatment, the association between PD‐L1 expression on tumor cells and clinical benefit from nivolumab remains poorly understood,^1^, ^2^ and no other reliable predictive markers have been identified. Although the high mutational burden is reported to be a potential biomarker,^5^, ^6^ its use in clinical care remains unfeasible. Accordingly, a routinely obtainable biomarker which identifies candidate patients for ICIs and predicts clinical response would be valuable.

Hematological inflammatory and nutritional markers might be predictive in patients with diverse histological types of neoplasia, including head and neck cancer.^7^, ^8^, ^9^, ^10^, ^11^, ^12^ Among these, well‐known examples include neutrophil‐to‐lymphocyte ratio (NLR) and the modified Glasgow prognostic score (mGPS). These are standardized markers and routinely available in daily practice. Evidence for a predictive role of inflammatory markers in patients treated with ICIs is increasing.^13^ Neutrophil‐to‐lymphocyte ratio is the most reported, and might therefore be predictive for ICI treatment in other types of carcinoma.^13^, ^14^, ^15^, ^16^, ^17^, ^18^ However, its predictive role in head and neck cancer has not been adequately studied. Additionally, to our knowledge, no report has found an association between mGPS and immunotherapy for any neoplasm. Given this, the possibility that routinely available inflammatory markers have a predictive role in patients with R/M SCCHN treated by nivolumab should be explored.

Here, we conducted a multicenter retrospective cohort study to investigate the impact of these hematological inflammatory markers on survival in patients with R/M SCCHN treated with nivolumab.

## 2 MATERIALS AND METHODS

### 2.1 Patients

This study was conducted under a retrospective cohort design in patients with R/M SCCHN treated with nivolumab at four hospitals from May 2017 to August 2018: Kitasato University Hospital, Tokyo Medical University Hospital, Tokyo Medical University Hachioji Medical Center, and International University of Health and Welfare Mita Hospital. One hundred and two consecutive patients were treated with nivolumab during this period. Among these, 2 patients without a history of platinum drug administration and 12 who were histologically diagnosed with non‐squamous cell carcinoma were excluded, leaving 88 patients for enrollment. The study was approved by the Institutional Ethics Review Board of each of these hospitals. With regard to consent to participate, patients could reject participation by opting‐out to an announcement on the institutions’ web sites. This study was performed in accordance with the Declaration of Helsinki.

### 2.2 Treatment and follow‐up

All cases were histologically diagnosed as squamous cell carcinoma in each institution and had been treated with platinum drug administration before treatment with nivolumab. Nivolumab was administered at 3 mg/kg every 2 weeks. Blood testing included complete blood cell counts, serum albumin (Alb), and serum C‐reactive protein (CRP) before each administration in all patients. After the start of the treatment, imaging examination by CT or MRI was performed every 4‐6 weeks, and response was evaluated using RECIST guideline version 1.1. Patients in whom nivolumab administration was terminated due to clinically obvious disease progression were judged as PD, even when image evaluation was not performed. Administration of nivolumab was also terminated in patients with unacceptable adverse events or at patient request.

### 2.3 Evaluation of markers

In this study, we evaluated NLR and mGPS using neutrophil count, lymphocyte count, CRP, and Alb in peripheral blood just before the start of nivolumab treatment. Neutrophil‐to‐lymphocyte ratio was calculated by division of absolute neutrophil and lymphocyte count and categorized into two groups by the optimum cutoff value against outcome, as described below. Regarding mGPS, patients with both an elevated CRP (>1.0 mg/dL) and decreased Alb (<3.5 g/dL) were assigned a score of 2; those with an elevated CRP (>1.0 mg/dL) and nondecreased Alb (≥3.5 g/dL) were assigned a score of 1; and those with a nonelevated CRP (≤1.0 mg/dL) were assigned a score of 0.

### 2.4 Statistical analysis

The primary endpoint of this study was overall survival (OS; interval between the date of first nivolumab treatment and the date of death from any cause or date of last follow‐up). Secondary endpoint was progression‐free survival [PFS; interval between the date of first nivolumab treatment and the date of diagnosis of progressive disease (PD), as evaluated and recorded by the attending physician], overall response rate [ORR: ratio of complete response (CR) or partial response (PR)], and disease control rate [DCR: ratio of CR, PR, or stable disease (SD)]. Participants who were lost to follow‐up were treated as censored. OS and PFS were estimated by the Kaplan‐Meier product‐limit method and tested by means of two‐sided log‐rank tests. To evaluate the survival impact of NLR and mGPS, we estimated hazard ratios (HRs) and 95% confidence intervals (95% CIs) using multivariate Cox proportional hazards models. ORR and DCR were compared by the stratified Cochran‐Mantel‐‐Haenszel method.

The main exposures of interest in this study were NLR and mGPS. First, we treated NLR as a continuous variable. In addition, to clarify the impact of NLR on survival and response, we dichotomized NLR by cutoff values evaluated using the area under the receiver operating characteristic curve (AUROC). We defined overall death as the objective standard and continuous values of NLR as the diagnostic test value.

Confounding variables considered in multivariate analyses were age (<65 vs ≥65), sex (male vs female), Eastern Cooperative Oncology Group performance status (ECOG PS) (0 vs 1‐2), primary tumor site (oral cavity, oropharynx, hypopharynx, larynx, nasal cavity, and others), metastasis (yes vs no), smoking (ever vs never), previous cetuximab use (yes vs no), previous chemotherapy regimen (1 vs ≥2), and institution [Tokyo Medical University (TMU) Hospital, Hachioji Medical Center of TMU, International University of Health and Welfare Mita Hospital (IUHWM), Kitasato University Hospital], treated by strata. Stratification was performed by dichotomized confounding variables, and interactions were assessed using interaction terms. All statistical analyses were performed using STATA version 14 (Stata Corp., College Station, TX, USA). All tests were two‐sided, and values of *P* < .05 were considered statistically significant.

## 3 RESULTS

### 3.1 Patients characteristics

Characteristics of the 88 patients are summarized in Table 1. Males were predominant (81%). Median age was 66.5 years (range, 23‐81 years). Regarding ECOG PS, about two‐thirds (67%) were 0. Primary tumor site was the nasopharynx in 10 cases (11%), oropharynx in 19 (22%), hypopharynx in 21 (24%), larynx in 10 (11%), oral cavity in 17 (19%), and other in 11 (13%). In 5 cases of double cancers, the more advanced was considered the primary tumor site (data not shown). Furthermore, 69% had a history of smoking, 59% had distant metastasis at the start of nivolumab treatment, and 66% had previous receipt of cetuximab. Regarding previous chemotherapy regimens, about 40% each had one or two regimens and 18% had three or more. No patients received Recombinant Human Granulocyte Colony‐stimulating Factor Injection within 2 weeks before nivolumab administration. Median follow‐up time was 6.1 months (range, 0.8‐16.9 months). Platinum refractoriness was seen in 67%, and surgery and radiotherapy were performed for 58% and 81%, respectively.

**Table 1:** Characteristics of patients at baseline

	Total	
	N = 88	(%)
Sex		
Male	71	(81)
Female	17	(19)
Age (y)		
<65	39	(44)
≥65	49	(56)
ECOG performance status^a^		
0	59	(67)
1	24	(27)
≥2	5	(6)
Primary site		
Nasopharynx	10	(11)
Oropharynx	19	(22)
Hypopharynx	21	(24)
Larynx	10	(11)
Oral cavity	17	(19)
Other^b^	11	(13)
Smoking		
Ever	61	(69)
Never	26	(30)
Unknown	1	(1)
With metastasis‐no.		
Previous receipt of cetuximab‐no.	52	(59)
Previous receipt of chemotherapy regimen‐no.	58	(66)
1	37	(42)
2	35	(40)
≥3	16	(18)
Institution		
Kitasato Uni.	41	(47)
Tokyo Medical Uni. (TMU)	15	(17)
Hachioji Medical Center of TMU	8	(9)
IUHW^d^	24	(27)
NLR		
Continuous (median, SD	5.4	(± 6.4)
High^c^	60	(68)
Low	28	(32)
mGPS		
0	41	(47)
1	25	(28)
2	22	(25)

Abbreviations: NLR, neutrophil/lymphocyte ratio; mGPS, modified Glasgow Prognostic Score.

^a^ Eastern Cooperative Oncology Group.

^b^ Others were nasal cavity (6), salivary gland (2), and ear (3).

^c^ Low and High: threshold of NLR was 7.0.

^d^ International University of Health and Welfare Mita Hospital.

Median OS and PFS were 9.5 months (95% CI, 7.9‐11.8) and 3.7 months (95% CI, 2.5‐5.09), respectively. Regarding clinical response as ORR and DCR, response to nivolumab treatment could not be evaluated in four cases due to insufficient time since the first nivolumab treatment were excluded from analysis. Finally, three cases (4%) were classified as CR, 10 (12%) as PR, 26 (31%) as SD, and 45 (54%) as PD.

### 3.2 Distribution of hematological inflammatory marker

Median NLR was 5.4 (± 6.4 standard deviation). The optimal cutoff value of NLR calculated by AUROC was 7.0, as shown in Table S1. Therefore, to clarify the impact of NLR on survival, we divided the patients into high‐ [NLR > 7, n = 60 (68%)] and low‐NLR groups [NLR < 7, n = 28 (32%)]. Regarding mGPS, 41 (47%), 25 (28%) and 22 (25%) patients were assigned a score of 0, 1, and 2, respectively. A Spearman's rank correlation test showed a weak positive correlation between NLR and mGPS (Spearman's rank correlation test; *r* = .313, *P* = .003).

### 3.3 Impact of hematological markers on overall survival

**Figure 1:**
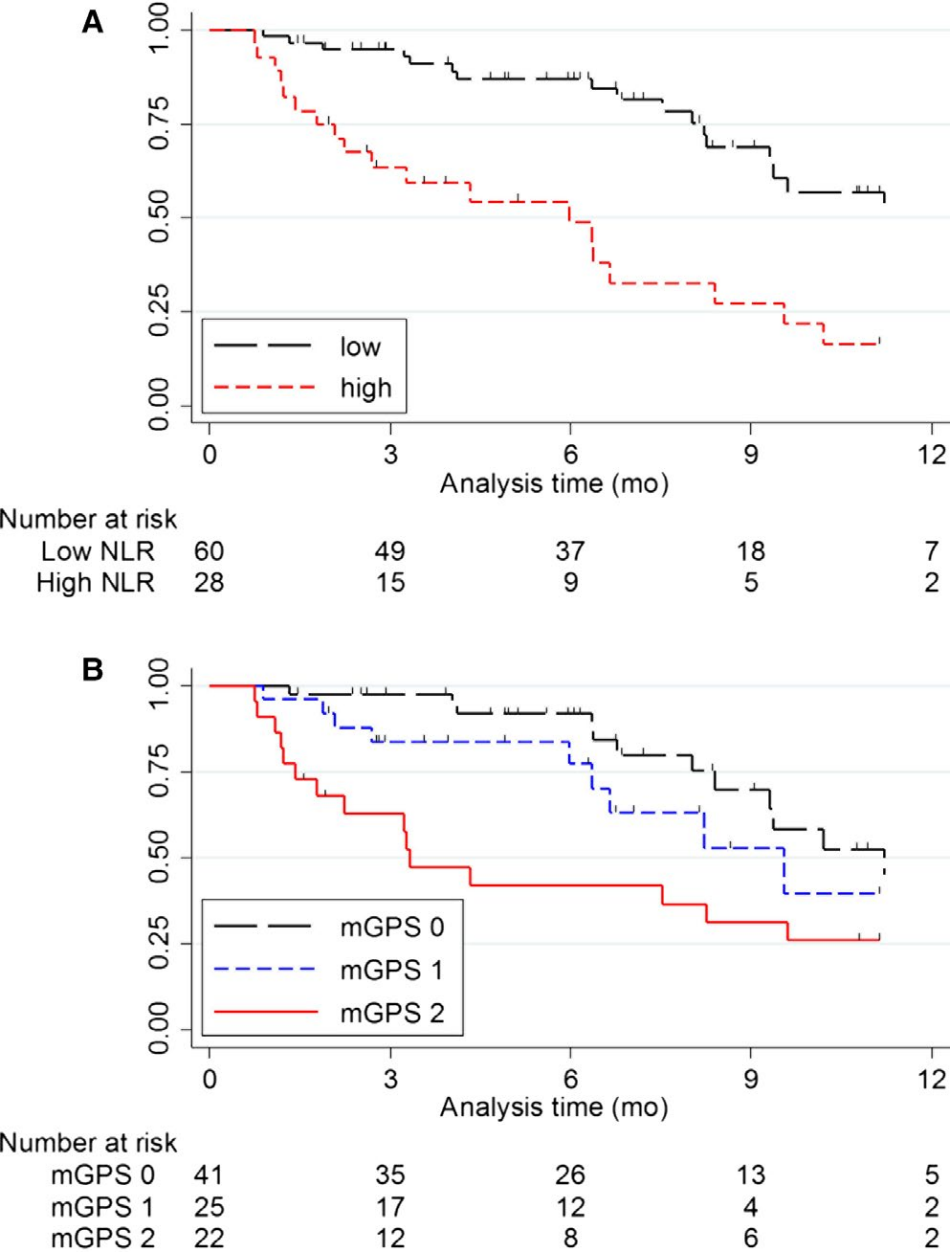
Kaplan‐Meier survival curves of NLR (A) and mGPS (B) on OS. A, Patients with high NLR (n = 28) had significantly worse OS than patients with low NLR (n = 60) (one‐year OS: 45.3% (95% CI, 25.5‐63.4) vs 16.3% (95% CI, 4.2‐35.4, *P*‐value < .001). B, Patients with mGPS of 2 (n = 22) had significantly worse OS than patients with mGPS of 0 (n = 41) (1‐year OS: 37.4% (95% CI, 15.5‐59.6) vs 26.1% (95% CI, 9.9‐46.3), *P*‐value = .004)

When NLR was treated as a continuous variable, higher NLR was significantly associated with worse survival (*P*‐value < .001, Table 2). After adjustment for potential confounders, elevated NLR was associated with a 17% increase in deaths (adjusted HR of continuous NLR = 1.17; 95% CI, 1.09‐1.26; *P*‐value < .001). When evaluated by dichotomized NLR, patients with a higher NLR had significantly worse OS than patients with a low NLR [1‐year OS: 45.3% (95% CI, 25.5‐63.4) vs 16.3% (95% CI, 4.2‐35.4), *P*‐value < .001 by the log‐rank test, Figure 1A]. This association remained consistent after adjustment for potential confounders [adjusted HR = 4.40; 95% CI, 1.78‐10.88; *P*‐value = .001; Table 2].

**Table 2:** Impact of inflammatory markers on overall survival and progression‐free survival

			Overall survival							
Inflammatory marker					Crude			Adjusted^a^		
	N = 88	(%)	1‐y OS (%)	(95% CI)	HR	(95% CI)	*P*‐value	HR	(95% CI)	*P*‐value
NLR										
Continuous					1.08	(1.03‐1.12)	.001	1.17	(1.09‐1.26)	<.001
Dichotomized										
Low^b^	60	(68)	45.3	(25.5‐63.4)	1 (reference)			1 (reference)		
High^b^	28	(32)	16.3	(4.2‐35.4)	3.45	(2.03‐11.57)	<.001	4.40	(1.78‐10.88)	.001
mGPS										
0	41	(47)	37.4	(15.5‐59.6)	1 (reference)			1 (reference)		
1	25	(28)	39.5	(12.1‐66.4)	1.49	(0.64‐3.50)	.358	1.87	(0.60‐5.81)	.280
2	22	(25)	26.1	(9.9‐46.3)	2.85	(1.35‐6.02)	.006	4.20	(1.54‐11.49)	.005
					*P*_trend_	<.001		*P*_trend_	.005	

			Progression‐free survival							
Inflammatory marker					Crude			Adjusted^†^		
			1‐y PFS (%)	(95% CI)	HR	(95% CI)	*P*‐value	HR	(95% CI)	*P*‐value
NLR										
Continuous					1.04	(1.00‐1.08)	.035	1.08	(1.03‐1.13)	.003
Dichotomized										
Low^b^	60	(68)	39.1	(19.1‐58.7)	1 (reference)			1 (reference)		
High^b^	28	(32)	9.0	(1.6‐24.8)	2.41	(1.39‐4.20)	.002	3.37	(1.64‐6.92)	.001
mGPS										
0	41	(47)	41.5	(24.3‐57.8)	1 (reference)			1 (reference)		
1	25	(28)	10.8	(1.0‐34.3)	1.80	(0.98‐3.41)	.056	2.92	(1.28‐6.69)	.014
2	22	(25)	24.8	(8.1‐46.1)	2.38	(1.20‐4.73)	.013	2.01	(0.87‐4.68)	.102
					*P*_trend_	.005		*P*_trend_	.022	

Abbreviations: mGPS, modified Glasgow Prognostic Score; NLR, neutrophil/lymphocyte ratio.

^a^ Adjusted for sex, age, smoking, performance status, primary site, previous cetuximab use, previous chemotherapy regimen, metastasis, and institution by strata.

^b^ Low and High: threshold of NLR was 7.0.

Regarding mGPS, elevated mGPS was associated with worse OS in univariate analysis (*P*_trend_ < .001). Patients with an mGPS score of 2 showed significantly worse OS than those with an mGPS of 0 [1‐year OS: 37.4% (95% CI, 15.5‐59.6) vs 26.1% (95% CI, 9.9‐46.3), *P*‐value = .004, Figure 1B]. This association remained consistent after adjustment for potential confounders [adjusted HR = 4.20 (95% CI, 1.54‐11.49), *P*‐value = .005, Table 2]. In addition, a significant dose‐response relationship was observed (*P*_trend_ = .005).

### 3.4 Impact of hematological markers on OS stratified by clinical covariates

**Figure 2:**
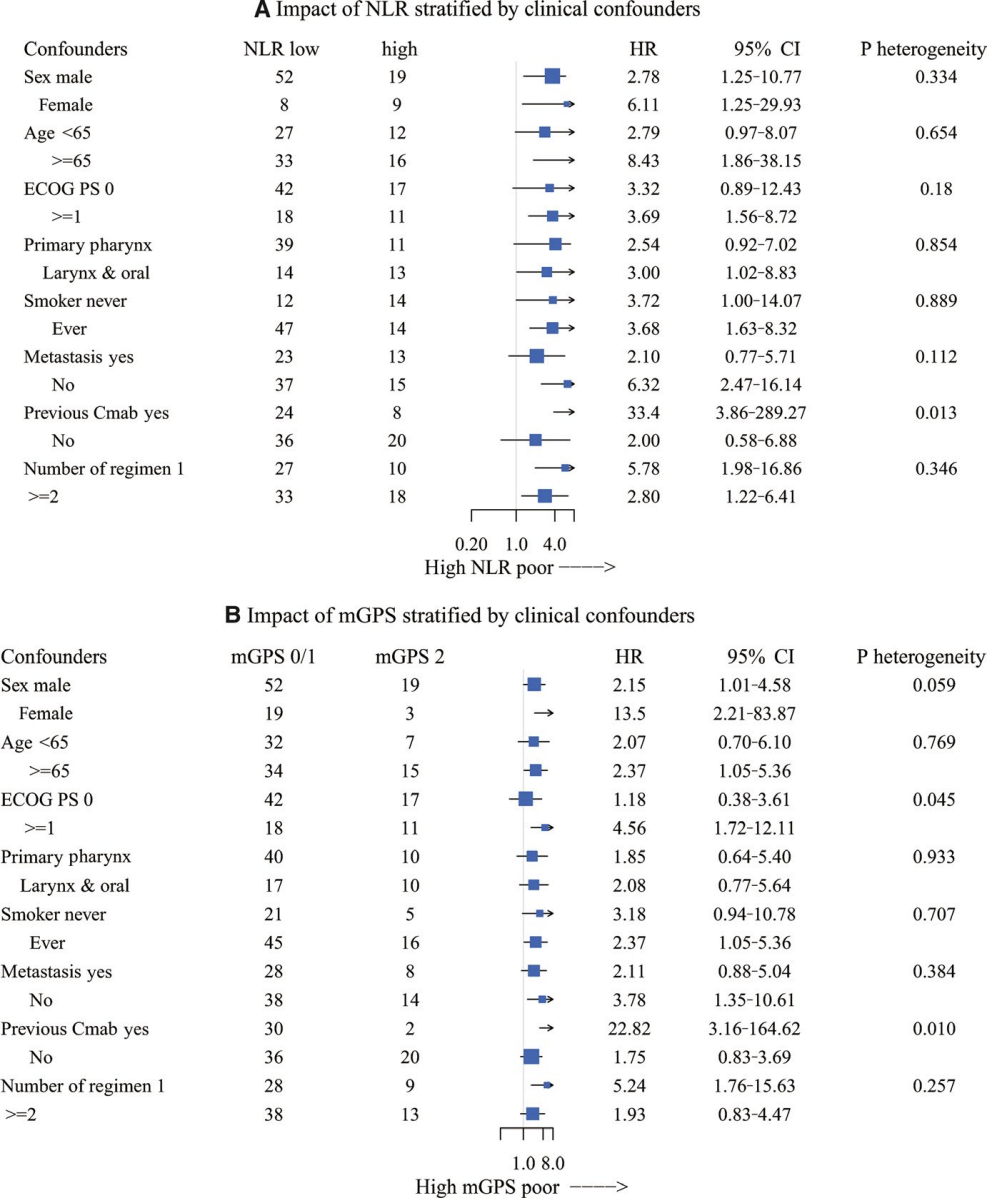
Hazard ratio for death of NLR (A) and mGPS (B) stratified by clinical covariates. A, High NLR showed consistently high HR for death compared to low NLR across all subgroups. B, mGPS of 2 showed a consistently high HR for death relative to mGPS 0‐1 across all subgroups. Interaction was observed between history of previous cetuximab use in both NLR and mGPS

To evaluate the consistency of the impact of hematological markers on OS, we evaluated the impact of NLR and mGPS on stratification by clinical covariates. With regard to NLR, the estimated HR for death on analysis of OS with high vs low NLR was more than 1 across all subgroups (Figure 2A). Similarly, an mGPS of 2 was consistently associated with worse survival than an mGPS of 0 or 1 in any subgroup (Figure 2B). For both NLR and mGPS, interaction was limited to patients with a history of cetuximab use.

### 3.5 Impact of hematological markers on progression‐free survival

**Figure 3:**
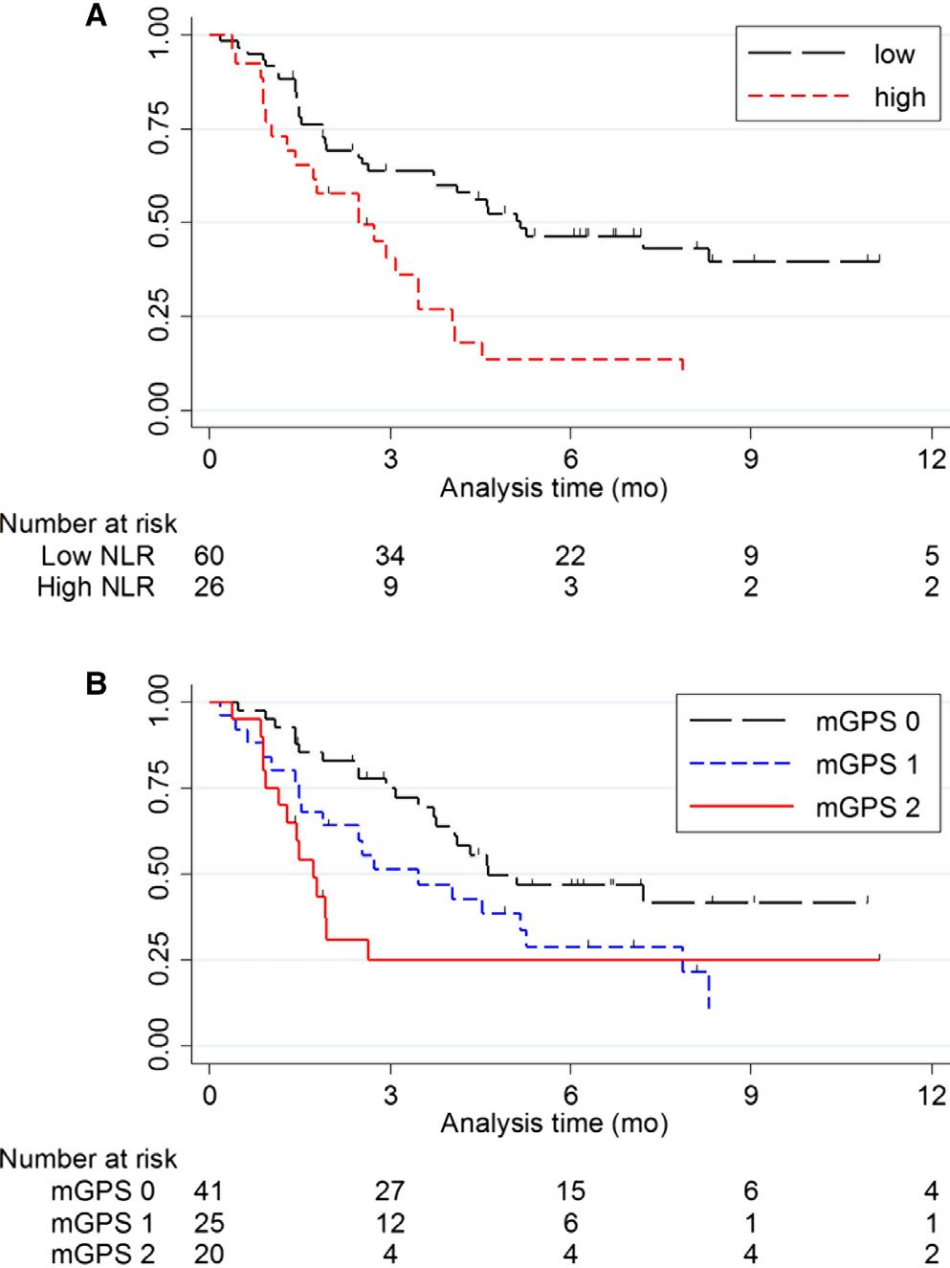
Kaplan‐Meier survival curves of NLR (A) and mGPS (B) on PFS. A, Patients with high NLR (n = 28) had significantly worse PFS than patients with low NLR (n = 60) (1‐year PFS: 39.1% (95% CI, 19.1‐58.7) vs 9.0% (95% CI, 1.6‐24.8), *P*‐value = .001). (B) Patients with mGPS of 2 (n = 22) had significantly worse PFS than patients with mGPS of 0 (n = 41) (1‐year PFS: 41.5% (95% CI, 19.1‐58.7) vs 24.8% (95% CI, 8.1‐46.1), *P*‐value = .007)

Similarly to OS, patients with a high NLR had a significantly higher HR for progression than patients with a low NLR, even after adjustment for potential confounders [1‐year PFS: 39.1% (95% CI, 19.1‐58.7) vs 9.0% (1.6‐24.8), *P*‐value = .001; adjusted HR = 3.37 (95% CI, 1.64‐6.92), *P*‐value = .001; Figure 3A and Table 2]. With regard to mGPS, patients assigned a score of 2 had worse PFS than those with 0, even after adjustment for potential confounders [1‐year PFS: 41.5% (95% CI, 24.3‐57.8) vs 24.8% (95% CI, 8.1‐46.1), *P*‐value = .007; adjusted HR = 2.01 (95% CI, 0.87‐4.68), *P*_trend_ = .022; Figure 3B and Table 2].

### 3.6 Impact of hematological markers on clinical response

Table 3 shows the association of hematological markers with clinical response, defined as ORR or DCR. With regard to NLR, the association between dichotomized NLR and ORR was not obvious [ORR: 17.8% (95% CI, 7.5‐28.2) vs 10.7% (95% CI, 1.4‐22.9), *P*‐value = .414]. However, compared to a low NLR, a high NLR was significantly associated with a lower DCR [57.1% (95% CI, 43.8‐70.5) vs 25.0% (95% CI, 7.9‐42.1), *P*‐value = .002)]. Similarly, although an association between ORR and mGPS was not apparent, an mGPS of 2 was significantly associated with a lower DCR compared to an mGPS of 0 or 1 [DCR 52.3% (95% CI, 39.7‐65.0) vs 28.6% (95% CI, 7.5‐49.6), *P*‐value = .038].

**Table 3:** Impact of inflammatory marker on overall response rate and disease‐control rate

Inflammatory marker			Overall response					Disease control				
	N = 84	(%)	CR/PR	SD/PD	*P*‐value^a^	ORR (%)	(95% CI)	CR/PR/SD	PD	*P*‐value^a^	DCR (%)	(95% CI)
NLR												
Low	56	(68)	10	46		17.8	(7.5‐28.2)	32	24		57.1	(43.8‐70.5)
High	28	(32)	3	25		10.7	(1.4‐22.9)	7	21		25.0	(7.9‐42.1)
					.414					.002		
mGPS												
0/1	63	(35)	10	53		15.9	(6.5‐25.1)	33	30		52.3	(39.7‐65.0)
2	21	(25)	3	18		14.3	(2.0‐30.6)	6	15		28.6	(7.5‐49.6)
					.922					.038		

Abbreviations: DCR, disease control rate; mGPS, modified Glasgow Prognostic Score; NLR, neutrophil/lymphocyte ratio; ORR, overall response rate.

^a^ Cochran‐Mantel‐Haenszel test stratified by age, sex, and previous Cmab use.

## 4 DISCUSSION

This study demonstrated that hematological inflammatory markers, namely NLR and mGPS, were significantly associated with clinical outcomes in patients with R/M SCCHN treated with nivolumab. Patients with higher NLR and mGPS showed significantly worse OS, even after adjustment for potential confounders. Similarly, these associations were consistent with PFS. In addition, both were significantly associated with DCR, but not with ORR. These results suggest that these hematological inflammatory markers are predictive markers of outcome in patients with R/M SCCHN treated with nivolumab.

NLR and mGPS, combinations of cell components and protein components obtained from peripheral blood, are well‐known indicators of systemic inflammation.^8^, ^9^ Increasing evidence suggests that among patients with any of several malignancies, including head and neck cancer, these were independent prognostic markers and predictor of the efficacy of chemotherapy.^7^, ^9^, ^10^, ^12^, ^19^ Furthermore, several reports have suggested that NLR might be a predictive marker in patients treated with ICIs for non‐small cell lung cancer, melanoma, and renal cell carcinoma.^13^, ^14^, ^15^, ^16^, ^17^, ^18^ Another recent paper suggested that NLR might be a predictor of outcome in patients with R/M SCCHN treated with nivolumab, albeit that patient numbers were small.^20^ These previous findings appear to be consistent with our present results. Meanwhile, the association between mGPS and survival in patients treated with nivolumab has remained unclear. To our knowledge, this is the first report to evaluate the survival impact of these hematological inflammatory markers simultaneously in patients with R/M SCCHN treated with nivolumab.

Although the mechanism of the association between these hematological inflammatory markers and clinical outcomes in patients with R/M SCCHN treated with nivolumab remains unclear, a number of possibilities can be proposed based on previous evidence. The systemic inflammatory response promotes tumor cell invasion by microvascular regeneration, tumor cell proliferation, and tumor metastasis.^10^, ^21^ Additionally, this response assists tumor cell differentiation and suppresses host immune cell activity.^10^, ^22^ As immune function plays an important role in treatment with ICIs, any factor affecting immunity may impact the response to this treatment.

Neutrophil‐to‐lymphocyte ratio is calculated by dividing neutrophil count by lymphocyte count. Neutrophils produce and stimulate the secretion of several tumor‐promoting growth factors that provide a favorable environment for tumor development and progression.^10^, ^16^ In particular, IL‐6 has been identified as promoting ICIs resistance.^23^, ^24^ Furthermore, an increase in the number of neutrophils surrounding cancerous tissue can suppress antitumor immune responses while activating T lymphocytes and natural killer cells.^10^ In contrast, lymphocytes are immune cells which are considered to exert antitumor effects. A decrease in lymphocytes, including cells of the innate immune system, suppresses the immune response ^25^, ^26^ and attenuates the antitumor specific immune system.^16^, ^27^, ^28^ Altogether, an increase in pretreatment NLR—namely an increase in neutrophils and/or decrease in lymphocytes—is considered to lead to the progression of tumors and a worse prognosis in patients treated with ICIs. Although the exact reason why the optimal cutoff value of the ROC curve was 7 is unclear, we speculate that most patients with R/M SCCHN might have had worse laryngeal function and an increase in neutrophil count due to aspiration,^29^ resulting in higher NLR. Furthermore, a similar trend was observed when NLR cutoff value was 5, as indicated in most other malignancies (data not shown).

In contrast, mGPS is based on a combination of elevated CRP and hypoalbuminemia. Like NLR, it is an indicator of underlying systemic inflammation and may be a surrogate marker of precancer cachexia, which worsens the prognosis of patients with cancer.^11^, ^19^, ^30^, ^31^ CRP is produced by the action of IL‐6 on hepatocytes. Therefore, it reflects the amount of IL‐6 in circulating blood, which has been identified as promoting ICIs resistance.^32^ In addition, elevated CRP accelerates angiogenesis based on increased vascular growth factors and interleukin, and leads to tumor progression.^19^, ^33^, ^34^ Hypoalbuminemia often occurs secondary to systemic inflammatory responses and is observed together with increasing CRP in patients with various types of cancer.^19^, ^35^, ^36^ Furthermore, an impaired nutritional status, reflected by hypoalbuminemia, reduces tolerance to adverse events and compliance with treatment.^19^, ^37^ Considered together, an increased mGPS score, which means elevated CRP and/or reduced Alb, is considered to lead to tumor progression and a worse prognosis in patients treated with ICIs.

Thanks to the collective effects of these mechanisms, elevated pretreatment NLR, and mGPS correlated with worse OS, PFS, and DCR in patients treated with nivolumab. In this study, NLR and mGPS were associated with DCR, but not with ORR. Most clinical studies investigating the association between the best response in immunotherapy and survival have reported that DCR is associated with prognosis, although it is not associated with ORR and prognosis.^38^, ^39^ Given this, our present finding that a higher score for these inflammation markers is related to a lower DCR, resulting in a worse prognosis, might be plausible.

This study suggests that high pretreatment values for NLR and mGPS—which are routinely obtained hematological parameters and systemic inflammation indicators—are predictive markers for immunotherapy with nivolumab. At the same time, we also considered the possibility that the effect of immunotherapy with nivolumab would be insufficient when systemic inflammation was high. Although nivolumab is now approved as palliative therapy for head and neck cancer, we considered it would be better to start administration before the establishment of a systemic inflammatory response and accompanying inhibition of the immune system due to tumor progression.

Our study has several methodological strengths. Since the clinician deciding the treatment had no information on the association between hematological marker and prognosis, information bias appears unlikely. In addition, eligible participants were selected in accordance with the inclusion criteria from among all patients with R/M SCCHN treated with nivolumab in multiple institutions, reducing the likelihood of selection bias. Furthermore, the analysis adjusted for potential confounders, including detailed clinical information such as previous cetuximab use.

Several limitations should also be addressed. First, the study was conducted under a retrospective design, and although conducted under a multicenter design, sample size is relatively small. Even with such a limited size, however, our findings showed a consistent direction in multivariable analysis and in any subgroup. Additionally, the multicenter design meant that our sample size was larger than that of a previous report of hematological markers for immunotherapy for head and neck cancer. Second, our information on hematological markers reflected pretreatment status only, and not posttreatment markers which might also predict clinical outcomes.^13^, ^14^, ^17^ Third, we could not completely remove the potential influence of markers of infectious diseases, inflammation other than that due to cancer, and the use of glucocorticoid hormones. Moreover, we did not assess prior radiotherapy history, which is a potential confounder of impact on the outcomes of the patients treated with nivolumab.^40^ However, the findings remained consistent after adjustment and stratification by previous radiotherapy history (Table S2).

In conclusion, this study identified a significant association of pretreatment NLR and mGPS with OS, PFS, and DCR in patients with R/M SCCHN treated with nivolumab. We conclude that pretreatment hematological inflammatory markers might be predictive markers in patients with R/M SCCHN treated with nivolumab. A larger study to validate this association is warranted.

## CONFLICT OF INTEREST

The authors declare no conflict of interest.

## AUTHOR CONTRIBUTIONS

TM: Conceptualization, study design, acquisition and analysis of data, and writing the original draft. IO: Conceptualization and interpretation of data. CF: Study design and interpretation of data. MS: Interpretation and analysis of data. DK: Analysis of data. HS: Acquisition of data. KT: Supervision and acquisition of data. TK: Acquisition of data. TO: Acquisition of data. YT: Interpretation of data. KM: Supervision and acquisition of data. GO: Study design. TY: Supervision, analysis of data, and project management. All authors contributed to the writing of the final manuscript and approved the manuscript to be published, and agree to be accountable for all aspects of the work in ensuring that questions related to the accuracy or integrity of any part of the work are appropriately investigated and resolved.

## Supporting information

Table S1‐S2

## Data Availability

The main data generated or analyzed in this study are included in this published article. The remaining detailed datasets are available from the corresponding author on reasonable request.
